# A pre‐existing coordinated inflammatory microenvironment is associated with complete response of vulvar high‐grade squamous intraepithelial lesions to different forms of immunotherapy

**DOI:** 10.1002/ijc.33168

**Published:** 2020-07-03

**Authors:** Ziena Abdulrahman, Noel F. C. C. de Miranda, Bart W. J. Hellebrekers, Peggy J. de Vos van Steenwijk, Edith M. G. van Esch, Sjoerd H. van der Burg, Mariette I. E. van Poelgeest

**Affiliations:** ^1^ Department of Gynaecology Leiden University Medical Center Leiden The Netherlands; ^2^ Department of Pathology Leiden University Medical Center Leiden The Netherlands; ^3^ Department of Medical Oncology, Oncode Institute Leiden University Medical Center Leiden The Netherlands; ^4^ Department of Gynaecology Haga Hospital Den Haag The Netherlands; ^5^ Department of Gynaecology Maastricht University Medical Center Maastricht The Netherlands; ^6^ Department of Gynaecology Catharina Hospital Eindhoven The Netherlands

**Keywords:** imiquimod, immune microenvironment, therapeutic vaccine, TLR7, vulvar HSIL

## Abstract

Immunotherapy of vulvar high‐grade squamous intraepithelial lesion (vHSIL) is investigated as an alternative for surgery, because of high comorbidity and risk of recurrence. Limited evidence exists on the role and composition of the immune microenvironment in current immunotherapeutic approaches for vHSIL. The vHSIL of 29 patients biopsied before treatment with imiquimod were analyzed by two multiplex seven‐color immunofluorescence panels to investigate the pre‐existing T‐cell and myeloid cell composition in relation to treatment response. The samples were scanned with the Vectra multispectral imaging system. Cells were automatically phenotyped and counted with inForm advanced image analysis software. Cell counts and composition were compared to that of vHSIL patients before therapeutic vaccination (n = 29) and to healthy vulva (n = 27). Our data show that the immune microenvironment of complete responders (CR) to imiquimod resembled the coordinated infiltration with type 1 CD4^+^ and CD8^+^ T cells and CD14^+^ inflammatory myeloid cells also found in healthy vulva. However, more CD8^+^ T cells and FoxP3^+^ regulatory T cells were present in CR. The lesions of partial responders (PR) lacked such a coordinated response and displayed an impaired influx of CD14^+^ inflammatory myeloid cells. Importantly, complete responses after imiquimod or therapeutic vaccination showed the same dependency on a pre‐existing coordinated type 1 T‐cell and CD14^+^ myeloid cell infiltration. In conclusion, a good clinical outcome after two different forms of immunotherapy for vHSIL is associated with the presence of a primary inflammatory process resulting in the coordinated influx of several types of immune cells which is then amplified.

## Abstract 2

**What's new?**

Premalignant vulvar high‐grade squamous intraepithelial lesions (vHSIL) are predominantly induced by human papilloma virus infection. The immune microenvironment in vHSIL and its role in immunotherapeutic approaches remain largely unknown. This study is the first to show that a complete clinical response in patients is associated with a pre‐existent coordinated influx of type 1 T cells and CD14^+^ myeloid cells, irrespective of the type of successful immunotherapy given (topical imiquimod therapy or therapeutic vaccination). This coordinated immune microenvironment closely resembles that of healthy vulvar tissue, suggesting that an impaired primary inflammatory process acts as an immune resistance mechanism in non‐complete responders.

**Abbreviations**
CD: cluster of differentiation
CR: complete responder
DC: dendritic cell
FFPE: formalin‐fixed paraffin‐embedded
HPV: human papilloma virus
ICR: immunological constant of rejection
MDSC: myeloid derived suppressor cell
NR: nonresponder
PR: partial responder
TLR: toll‐like receptor
Treg: regulatory T cell
uVIN: usual vulvar intraepithelial neoplasia
vHSIL: vulvar high‐grade squamous intraepithelial lesion

## 1 INTRODUCTION

Vulvar high‐grade squamous intraepithelial lesion (vHSIL, previously known as usual vulvar intraepithelial neoplasia, uVIN) is a premalignant lesion predominantly induced by high‐risk human papillomavirus type 16 (HPV16). The current standard of care is surgery, which is associated with high comorbidity and recurrence, or topical application of the toll‐like receptor 7 (TLR7) agonist imiquimod to trigger innate immune responses.^1^, ^2^ In addition, new approaches are being investigated, aiming at either targeting the virus, using cidofovir,^1^ or by strengthening the immune system through systemic therapeutic vaccination to increase T‐cell reactivity against the viral oncoproteins E6 and E7. So far, complete responses have been observed in 35‐46% of patients treated with imiquimod,^1^, ^2^ 46% of patients treated with cidofovir^1^ and 28% of vHSIL patients treated with therapeutic vaccination.^3^

Imiquimod is thought to act on macrophages and dendritic cells (DCs), thereby stimulating the local production of pro‐inflammatory cytokines such as IFNγ, TNFα and IL‐12 which may steer the immune microenvironment towards a milieu that fosters the development of type 1 and cytolytic T‐cell responses (reviewed in Reference 4). In addition, imiquimod may interfere with the adenosine receptor signaling pathways, alleviating local suppression and boosting the pro‐inflammatory response (reviewed in Reference 5). Last but not least, it may also exert a pro‐apoptotic effect on tumor cells, through caspase activation.^6^ Studies on the effects of imiquimod on the immune microenvironment of vHSIL revealed imiquimod‐induced consistent increases in the numbers of CD8^+^ T cells, particularly in responding vHSIL patients.^2^, ^7^, ^8^ Increases in CD4^+^ T cells^7^ and CD1a^+^ DCs^2^ were also reported,^2^, ^7^ but not confirmed.^8^ Nonresponsiveness was associated with increased numbers of regulatory T cells (Tregs) after treatment.^7^, ^8^

We recently used two multispectral immunofluorescence panels, one for T cells (CD3, CD8, FoxP3, Tim3, Tbet, PD‐1 and DAPI) and one for myeloid cells (CD14, CD33, CD68, CD11c, CD163, PD‐L1 and DAPI) to show that a complete regression of vHSIL after therapeutic HPV16 peptide vaccination was associated with a coordinated pre‐existing pro‐inflammatory immune microenvironment comprising type 1 CD4^+^ and CD8^+^ T cells and CD14^+^ inflammatory macrophages.^9^ In contrast, studies on the pre‐existing immune infiltrate before imiquimod treatment showed that the numbers of vHSIL infiltrating CD4^+^ and CD8^+^ T cells as well as CD1a^+^ dendritic cells and CD68^+^ macrophages were not predictive for responsiveness.^7^, ^8^ In addition, while the number of pre‐existing Tregs was a negative predictor in one study,^7^ it was not in another.^8^ Notably, most studies identified immune cells at the single marker level, and some at a double or triple marker level. This provides only limited insights in the complexity and functional status of the many different immune cells that together compose the immune microenvironment, when compared to the multispectral panels that can be used nowadays.^9^

This prompted the questions if a more in‐depth study of the pre‐existing immune microenvironment composition can reveal an immune profile related to response to imiquimod therapy and whether such a pre‐existing immune profile in vHSIL differs from those vHSIL patients responding to therapeutic vaccination. Therefore, we studied the pre‐treatment immune microenvironment of 29 vHSIL patients in relation to the clinical response after imiquimod treatment, and compared this to the pre‐existing immune profile found in patients treated with a therapeutic HPV vaccine. Our data showed that a good clinical response to immunotherapy, being either imiquimod or therapeutic vaccination, is associated with a pre‐existing pro‐inflammatory coordinated immune microenvironment, characterized by CD14^+^ inflammatory cells, CD4^+^Tbet^+^ T cells and CD8^+^ T cells, and suggests that neither therapy is capable of overcoming an immunological cold microenvironment.

## 2 MATERIALS AND METHODS

### 2.1 Patient samples

Pretreatment formalin‐fixed paraffin‐embedded (FFPE) biopsies of 29 women of ≥18 years old with histologically confirmed vHSIL were retrospectively included. Patient samples were collected from two hospitals in the Netherlands: the Leiden University Medical Center (LUMC, Leiden, The Netherlands), and the Haga Hospital (the Hague, The Netherlands). Clinical complete response (CR, n = 14) upon therapy was defined as 100% lesion clearance, partial response (PR, n = 12) as ≥50% lesion clearance, and no response (NR, n = 3) as <50% lesion regression. Moreover, FFPE healthy HPV‐negative vulvar tissue from 27 anonymized women who underwent labia reduction surgery was included.

### 2.2 Multiplex immunofluorescence imaging

Two seven color multispectral immunofluorescence panels were applied, one for T cells, consisting of CD3, CD8, FoxP3, Tim3, Tbet, PD‐1, DAPI, and one for myeloid cells, consisting of CD14, CD33, CD68, CD11c, CD163, PD‐L1, DAPI, as previously published in.^9^ In these panels, a combination of direct detection (primary antibody directly labeled with fluorochrome) and indirect detection (fluorochrome labeled secondary antibody) of markers was used,^10^ and dim markers (PD‐L1, PD‐1 and Tbet) were tyramide signal amplified with Opal (PerkinElmer, Waltham, Massachusetts) to enable their detection by fluorescence microscopy. This combination of different marker detection methods, fully optimized for each marker, enabled the selection of the best primary antibodies without being restricted by clashing isotypes and species. Antibody specificity was first assessed with immunohistochemistry and monoplex immunofluorescence, tonsil slides serving as positive control, and subsequently the immunofluorescence detection conditions for each marker in multiplex immunofluorescence were optimized. A frequently encountered problem in multiplex immunofluorescence is spectral overlap between signals. To overcome this, we designed our panels in such a way that markers with an equally strong signal intensity were put in the same microscope detection filter, we alternated nuclear and membranous markers in the light spectrum when possible, and finally we found that alternating secondary Alexa fluorochromes (ThermoFisher Scientific, Waltham, Massachusetts) with CF dyes (Biotium, Hayward, California) when spectral overlap between two consecutive Alexa fluorochromes was detected despite the minimal exposure times used, resolved the cross bleed of signal successfully. An overview of the antibodies and staining methods included in each panel are shown in Table S1. In short, 4 μm FFPE tissue sections were deparaffinized, endogenous peroxidase was blocked with hydrogen peroxide and heat induced epitope retrieval was performed in the T‐cell panel with citrate (10 mM, pH 6.0) and in the myeloid cell panel with tris‐EDTA (10 mM/1 mM, pH 9.0). SuperBlock (ThermoFisher Scientific) was used to block nonspecific binding sites. First, the antibodies detected by Opal were applied, followed by the unconjugated antibodies which incubated overnight. On the second day, after binding of the previous with their corresponding fluorescently labeled secondary antibodies, the directly labeled primary antibodies were incubated for 5 hours, and finally DAPI was applied as nuclear counterstain.^9^

### 2.3 Quantification of immune cells in the TME

As published in Reference 9, immunofluorescence images of the entire tissue sections were acquired with the Vectra 3.0.5 multispectral imaging microscope (PerkinElmer) at ×20 magnification. Immune cells in the TME were automatically phenotyped and counted with inForm 2.4 image analysis software (PerkinElmer) after manual training. The software was trained to segment epithelium and stroma, segment DAPI+ nucleated cells, and assign a phenotype to each cell. All phenotypes were visually inspected on accurateness, and if errors were detected the training was further optimized until all discrepancies were resolved. Given the multitude of possible co‐expressed markers and the limited trainability of the inForm software in reliably detecting complex multi‐marker expressing cell phenotypes, each seven‐color panel was divided into multiple subanalyses that contained a small number of markers which the inForm software could robustly identify (Table S2). The phenotypes of all subanalyses were merged per cell based on its X,Y‐positions with an R script, which enabled the description of the full seven marker expression profile for each cell. Immune cell counts were normalized for tissue size (cells/mm^2^ epithelium and cells/mm^2^ stroma). After merging the subanalyses, a threshold of a median cell count ≥10 cells/mm^2^ in at least one group (NR, PR, CR or healthy vulva) was applied to study the changes in biologically common phenotypes.

### 2.4 Statistical analysis

Statistical data analysis was performed with SPSS 25.0 (IBM Corporation, Armonk, New York). The median immune counts of the different groups (PR, CR and healthy vulva) were compared to the nonparametric Mann‐Whitney *U* test. NRs were not included in the statistical analyses due to their small sample size (n = 3). Pearson correlation was used to study correlations between the T‐cell infiltrate and the myeloid cell infiltrate in each group. Two sided *P*‐values <.05 were marked as significant. GraphPad Prism 8.0.1 (GraphPad Software Inc., La Jolla, California) was used to create graphs.

## 3 RESULTS

### 3.1 The pretreatment immune microenvironment reveals high heterogeneity between vHSIL patients

**FIGURE 1:**
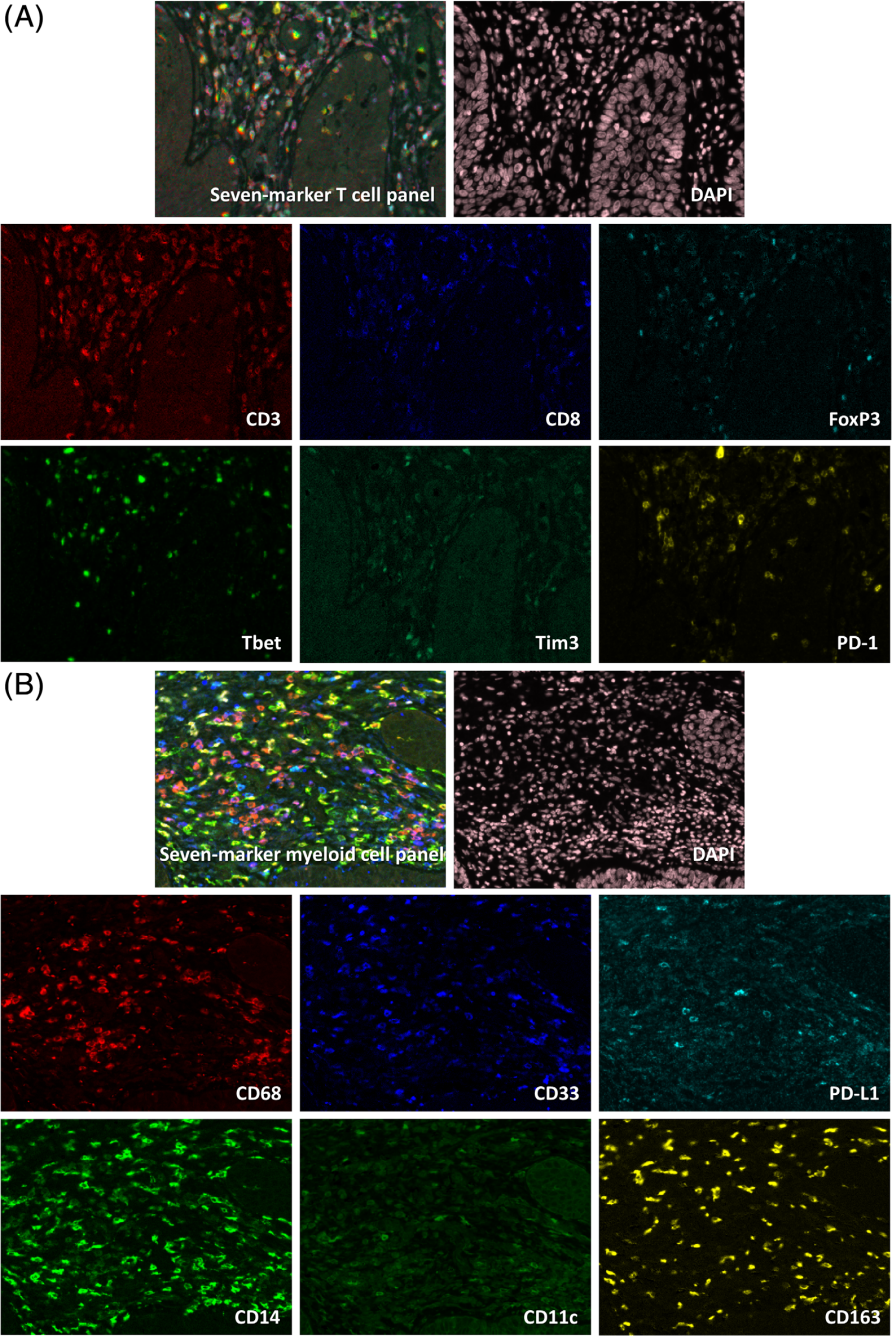
Multiplex immunofluorescence to detect T cells and myeloid cells in vHSIL. A, Composite image including all individual markers of the seven‐color T‐cell panel staining, consisting of the markers CD3, CD8, FoxP3, Tbet, Tim3, PD‐1 and DAPI. B, Composite image including all individual markers of the seven‐color myeloid cell panel staining, consisting of the markers CD33, CD68, CD163, PD‐L1, CD14, CD11c and DAPI

**FIGURE 2:**
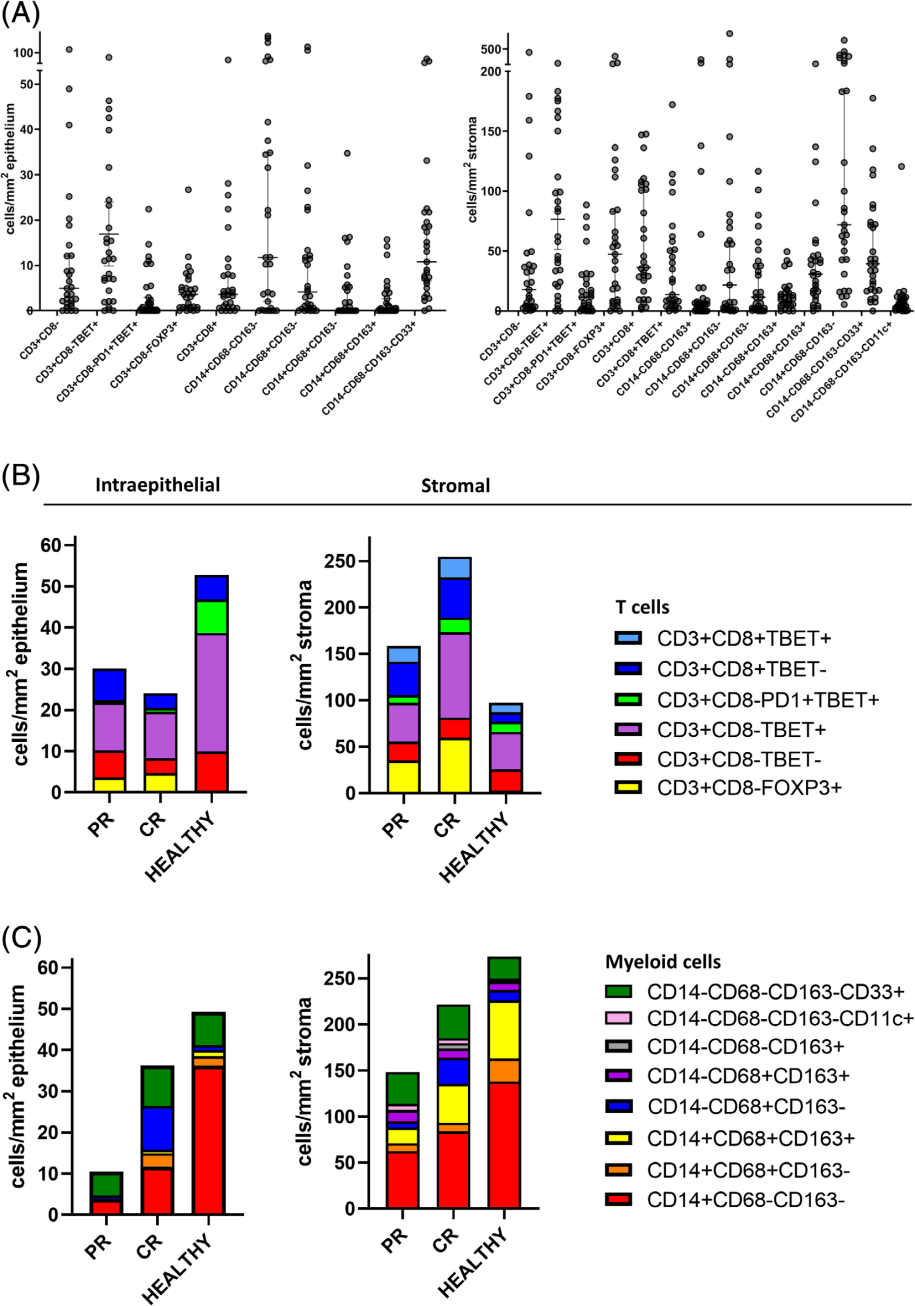
The pre‐imiquimod TME differs between the different response groups. A, The numbers of intraepithelial and stromal infiltrating T‐cell and myeloid cell subtypes are presented as cells/mm^2^ epithelium and cells/mm^2^ stroma in the pre‐imiquimod vHSIL biopsies of 29 patients. Each dot represents an individual sample, the horizontal bars indicate the median cell counts and the vertical bars are the 95% confidence intervals. B, T‐cell and C, myeloid‐cell infiltrate of the pre‐imiquimod vHSIL patients when grouped according to response (PR, CR), and compared to healthy vulvar tissue (n = 27), presented as median cells/mm^2^ epithelium and median cells/mm^2^ stroma. A threshold of a median cell count ≥10 cells/mm^2^ in at least one group (NR, PR, CR or healthy vulva) was applied to study the changes in biologically common phenotypes. CR, complete responders (n = 14); PR, partial responders (n = 12)

We analyzed the complexity of the vHSIL‐infiltrating T‐cell and myeloid cell populations in 29 patients (Table 1), before topical imiquimod treatment, using two seven‐color multiplex immunofluorescence panels (Tables S1 and S2). Analyses of these images stained for T cells and myeloid cells (Figure 1 and Figures S1‐S3) revealed six different phenotypes of T cells and eight different phenotypes of myeloid cells (Figure 2A). The CD3^+^CD8^−^ T cells detected were designated as CD4^+^ T cells, based on our earlier study showing that on average 99% of the CD3^+^CD8^−^ T cells were CD4^+^ T cells.^9^ Moreover, it showed that the vHSIL patients formed a heterogeneous group with high interpatient variability in the numbers of intraepithelial and stromal immune cells (Figure 2A and Table S3).

**TABLE 1:** Characteristics of vHSIL patients treated with topical imiquimod therapy

Patient number	Center	Age at start imiquimod	Response
1	LUMC	52	CR
2	LUMC	42	CR
3	LUMC	52	CR
4	LUMC	31	PR
5	LUMC	36	PR
6	LUMC	30	CR
7	LUMC	53	PR
8	LUMC	55	PR
9	LUMC	46	CR
10	LUMC	45	CR
11	LUMC	60	PR
12	LUMC	49	PR
13	LUMC	70	CR
14	LUMC	58	CR
15	LUMC	69	PR
16	LUMC	58	PR
17	LUMC	37	NR
18	HAGA	34	PR
19	HAGA	56	CR
20	HAGA	50	CR
21	HAGA	51	CR
22	HAGA	60	CR
23	HAGA	27	PR
24	HAGA	36	CR
25	HAGA	55	CR
26	HAGA	58	PR
27	HAGA	55	PR
28	HAGA	30	NR
29	HAGA	31	NR

Abbreviations: CR, complete responder, defined as 100% lesion clearance (n = 14); NR, nonresponders, defined as <50% lesion regression (n = 3); PR, partial responder, defined as ≥50% lesion clearance (n = 12).

### 3.2 Differences in the myeloid cell compartment of vHSIL correlate with response to imiquimod

The vulva is a highly immune active anatomical location as shown by the high numbers of T cells and myeloid cells infiltrating this healthy tissue (Figure 2). To study if quantitative or qualitative differences in immune infiltrate were associated with a different clinical outcome in vHSIL, we grouped the patients by clinical response (PR, CR) and compared them to healthy tissue. This revealed that while the PR and CR patients displayed abundant T‐cell infiltration in the stroma, with higher numbers of CD8^+^ T cells and CD3^+^CD8^−^FoxP3^+^ Tregs, the intraepithelial T‐cell infiltration was considerably reduced as compared to healthy tissue, lacking CD3^+^CD8^−^PD1^+/‐^Tbet^+^ activated type 1 T helper cells in particular (Figure 2B and Table S3). No overt differences in T‐cell infiltration existed between the PR and CR patients (Figure 2B and Table S3). The myeloid cell population differed the most among the different clinical groups. There was substantial intraepithelial and stromal infiltration by CD33^+^ immature myeloid cells (Figure 2C and Table S3), which most likely are myeloid derived suppressor cells (MDSC) based on our earlier studies,^11^ but their numbers did not differ between the groups. Furthermore, a substantial fraction of the myeloid cells in the stroma comprised different populations of CD14^+^ inflammatory myeloid cells, in particular CD14^+^CD68^−^CD163^−^ cells and CD14^+^CD68^+^CD163^+^ M2 macrophages. Whereas the numbers of the different stromal CD14^+^ inflammatory myeloid cell subpopulations between healthy vulva and CR lesions did not differ much, the numbers of these myeloid cell subpopulations were significantly lower in the lesions of PR patients (Figure 2C and Table S3). Finally, the number of stromal CD11c^+^ DCs was slightly increased in vHSIL when compared to healthy vulva.

Thus, while the numbers and composition of infiltrating immune cells of CR patients most closely resembled that of healthy vulva, PR patients showed impaired infiltration with inflammatory myeloid cells (Figure 2).

### 3.3 A disconnection between CD8 ^+^ T‐cell and myeloid cell infiltration in partial responders

**FIGURE 3:**
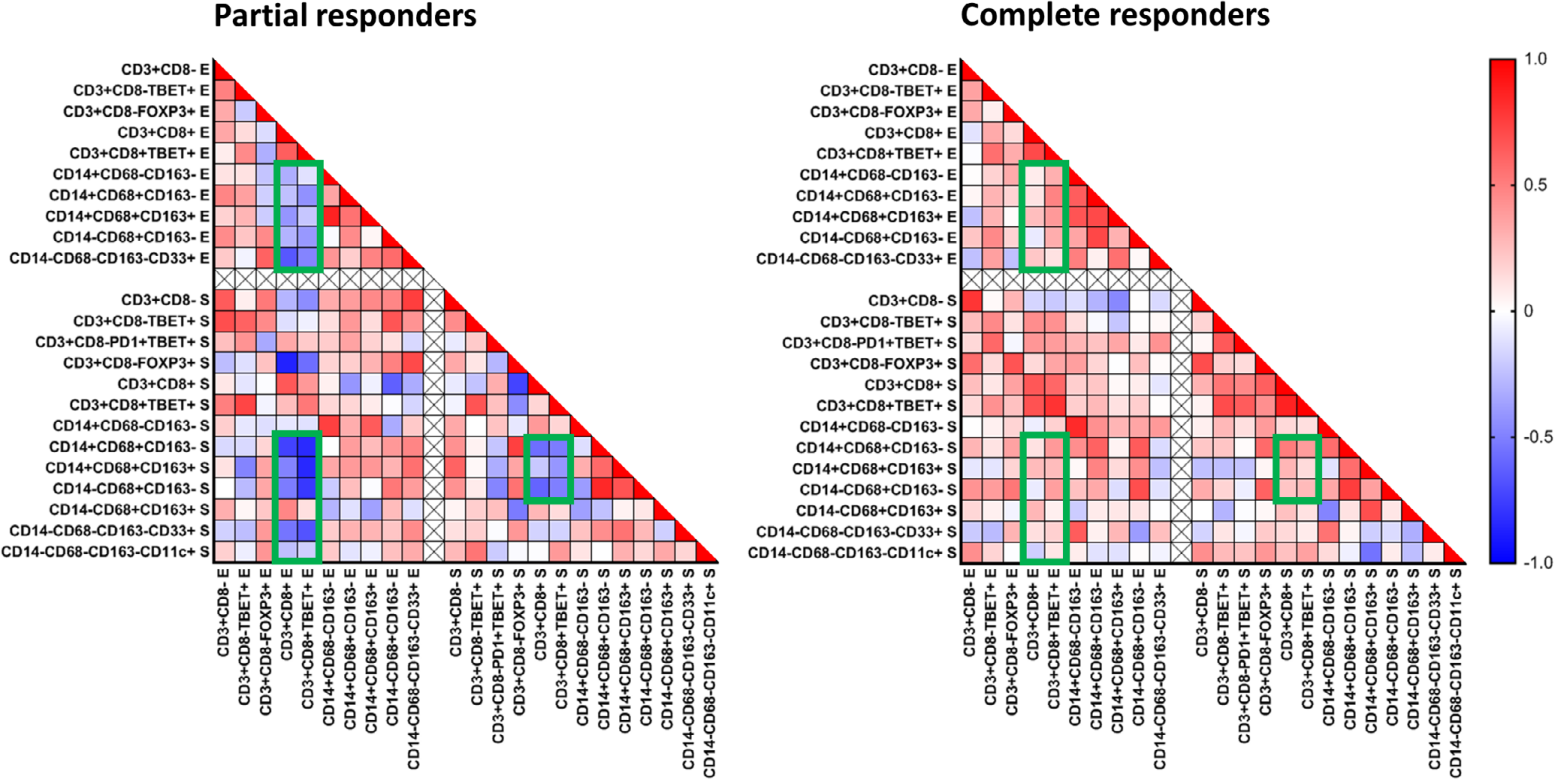
Correlation between the absolute numbers of tissue infiltrating T cells and myeloid cells in the pre‐imiquimod vHSIL biopsies of partial responders and complete responders. Nonparametric Spearman *r* correlation analysis (two‐tailed) was performed to analyze the co‐infiltration of the indicated different immune cell subtypes in the epithelium (E) and stroma (S), and is shown in a heatmap for vHSIL partial responders and complete responders to topical imiquimod treatment. Red indicates a strong positive correlation between the two cell phenotypes, blue a strong negative correlation, the green squares indicate the most striking differences between the partial and complete responders. A threshold of a median cell count ≥10 cells/mm^2^ in at least one group (NR, PR, CR or healthy vulva) was applied to study the changes in biologically common phenotypes. CR, complete responders (n = 14); PR, partial responders (n = 12)

Coordination of the immune response plays an important prognostic role in many cancers.^12^ To assess how the infiltration of the various T cells and myeloid cells was connected, we used Spearman *r* correlation and plotted the correlation graphs for the groups of PR and CR patients (Figure 3). The patients with a CR after imiquimod treatment showed a general well‐coordinated infiltration shown by the positive correlations between type 1 (defined as Tbet^+^) CD4^+^ and CD8^+^ T cells as well as the different types of myeloid cells. This was not only observed in the epithelial and stromal compartments but also between these compartments (Figure 3). In contrast, the vHSIL of patients with a PR after imiquimod treatment displayed a strong negative correlation between infiltrating CD8^+^ T cells (irrespective of Tbet expression) and the majority of myeloid cell subtypes, and this disconnection was observed in both the intraepithelial and stromal compartments (Figure 3).

### 3.4 The composition of the vHSIL immune microenvironment in complete responders fits best with the mode of action of the chosen immunotherapeutic treatment

**FIGURE 4:**
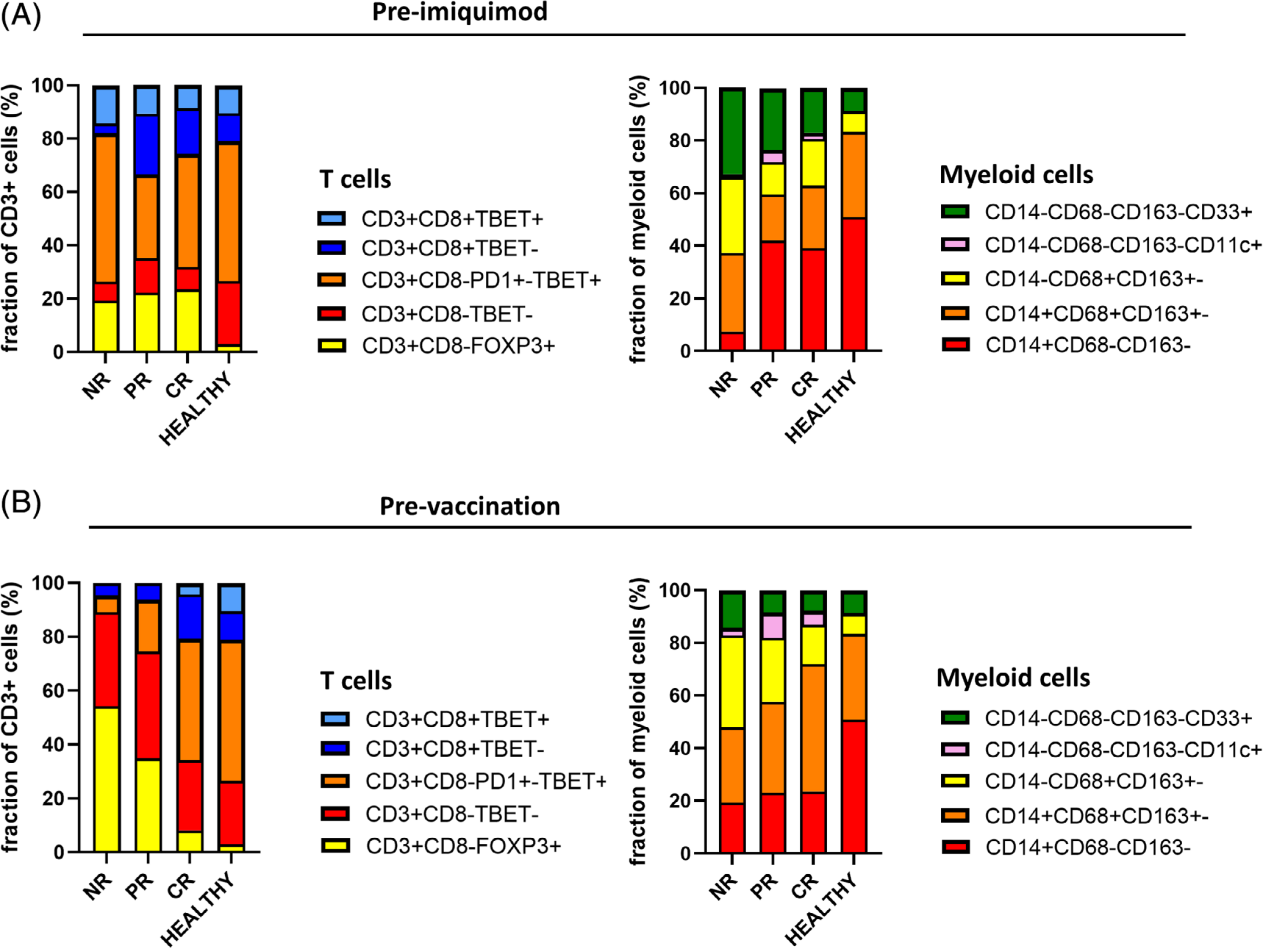
Fractional differences between the pre‐imiquimod and pre‐vaccination immune microenvironment in vHSIL, divided based on clinical response to the respective therapy. A, Fractional composition of the T cells and myeloid cells in the pre‐imiquimod vHSIL cohort, grouped according to response (NR: n = 3, PR: n = 12, CR: n = 14), and compared to healthy vulva (n = 27). B, Fractional composition of the T cells and myeloid cells in the pretherapeutic vaccination vHSIL cohort, grouped according to response (NR: n = 12, PR: n = 10, CR: n = 7), and compared to healthy vulva (n = 27)

**FIGURE 5:**
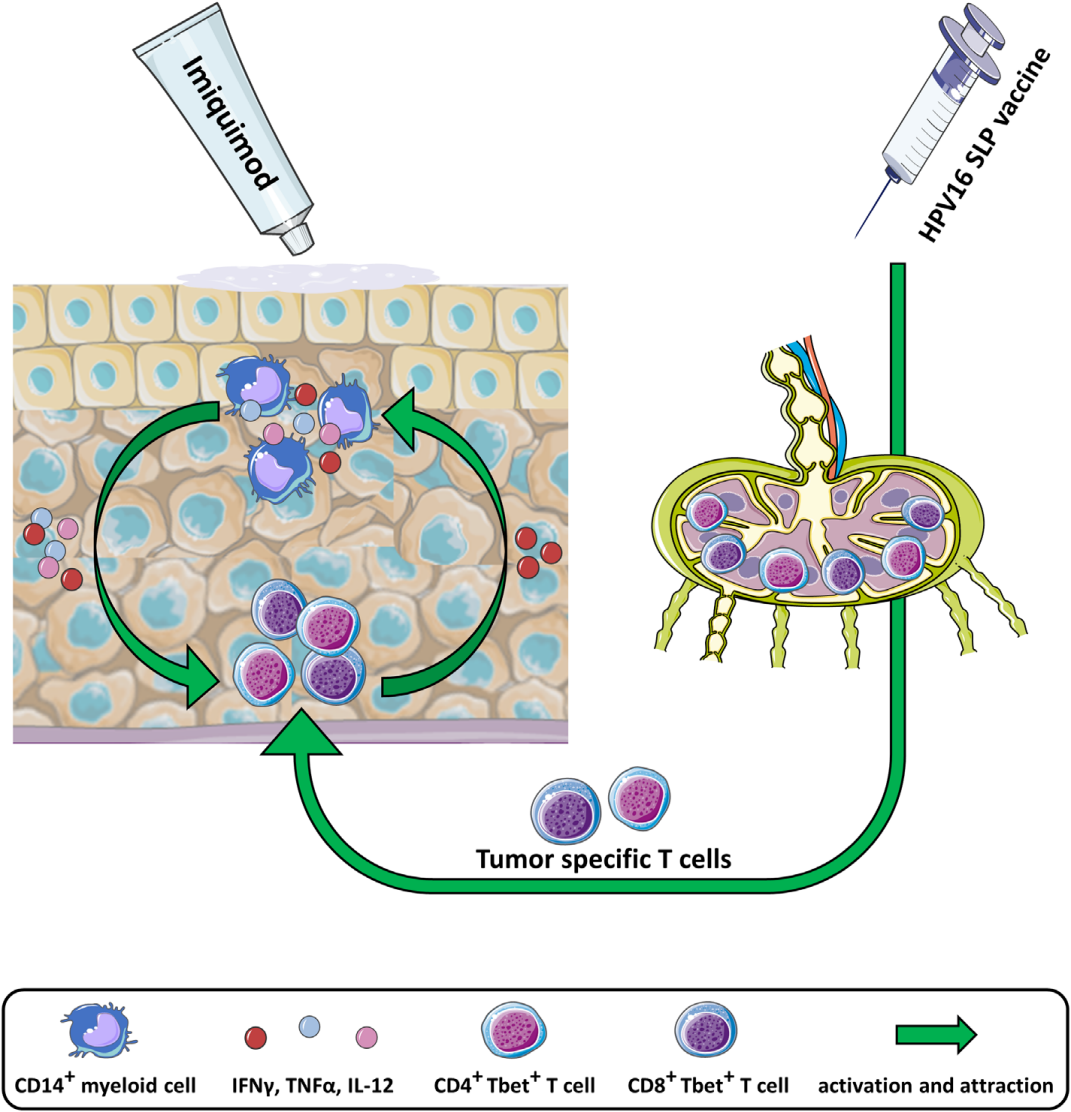
Summary of complete responder's vHSIL immune microenvironment in the context of the mode of action of imiquimod and therapeutic vaccination. The vHSIL immune microenvironment of complete responders to imiquimod and therapeutic HPV16 synthetic long peptide (SLP) vaccination comprises a coordinated infiltration with type 1 (Tbet^+^) CD4^+^ and CD8^+^ T cells as well as CD14^+^ inflammatory myeloid cells. Imiquimod stimulates CD14^+^ myeloid cells to produce pro‐inflammatory cytokines which activate and attract the type 1 T cells.^13^ Whereas, therapeutic vaccination stimulates the type 1 T‐cell response, which in turn produces cytokines that also stimulate the influx of the lesion by CD14^+^ myeloid cells.^9^ This figure was created using adapted images of Servier Medical Art, licensed under a creative commons attribution 3.0 unported license

The correlation between a CR after imiquimod and a pre‐existent high infiltration of the lesions with CD4^+^Tbet^+^ T cells and CD14^+^ inflammatory myeloid cells (Figure 2) as well as a coordinated overall immune cell infiltration (Figure 3) was also observed by us in vHSIL patients treated with a therapeutic vaccine containing synthetic long peptides of HPV16 E6 and E7 oncoproteins.^9^ This suggests that the immune composition of the lesions of CR patients is highly similar irrespective of the immunotherapeutic treatment. We, therefore, compared the composition of the stromal T‐cell and myeloid cell compartment in both groups of treated patients. Interestingly, the composition of the T‐cell fractions did not overtly differ between the groups of patients with a different clinical outcome after imiquimod treatment (Figure 4A), whereas this was clearly the case between the groups of patients treated with a therapeutic vaccine (Figure 4B). Interestingly, the myeloid cell composition did not differ much between the therapeutically vaccinated patient response groups, contrary to the different response groups to topical imiquimod treatment. In the latter, patients with a PR or CR had a considerably different myeloid cell composition compared to NR patients (Figure 4), implying that for response to imiquimod the number and composition of vHSIL infiltrating myeloid cells is of importance. Thus, whereas the composition of the T‐cell compartment was important for the outcome of a therapy aiming to improve the T‐cell response by therapeutic vaccination, the numbers of CD14^+^ cells in the myeloid cell compartment were more important for response to a treatment with topical imiquimod, known to stimulate myeloid cells^4^ and CD14^+^ cells in particular^13^ (Figure 5).

## 4 DISCUSSION

Here we show that a complete regression of vHSIL after imiquimod treatment is associated with a pre‐existing well‐coordinated overall infiltration between T cells and myeloid cells, and in particular the influx of vast numbers of vHSIL‐infiltrating type 1 T cells and CD14^+^ inflammatory myeloid cells. Healthy vulva is richly infiltrated and guarded by pro‐inflammatory T cells and myeloid cells. While the immune cell infiltration in the group of patients with a CR did not differ much from healthy vulva, the myeloid cell infiltration of lesions from PR patients was significantly lower than in healthy vulva. This was also visualized by the clear disconnection between the infiltration with CD8^+^ T cells and myeloid cells in the group of patients with a PR. The requirement for an ongoing T‐cell response for imiquimod to be effective was also previously reported^14^ but here it is shown that also a strong influx with CD14^+^ myeloid cells should be maintained. This would be in line with the mode of action of imiquimod which is acting predominantly on CD14^+^ cells,^13^ the activation of which leads to the increased infiltration of several types of adaptive immune cells.^2^, ^7^, ^8^ The presence of these CD14^+^ cells at lower numbers and in an unfavorable balance with suppressive immune cells (M2 macrophages, MDSC) may serve as a primary resistance mechanism to imiquimod treatment.

In our recent study on the immune microenvironment of vHSIL completely regressing after therapeutic vaccination, we also found a well‐coordinated immune infiltration in CR patients, and a positive association between clinical efficacy and high numbers of vHSIL‐infiltrating type 1 T cells and CD14^+^ inflammatory myeloid cells. Moreover, the vHSIL that led to a PR after vaccination displayed a disconnection between CD8^+^ T‐cell and myeloid cell infiltration.^9^ However, as shown in the current study, the clinical outcome after therapeutic vaccination was more related to differences in the composition of the CD4^+^ T‐cell fraction, fitting with the mode of action of this vaccine.^3^, ^15^, ^16^, ^17^

The combination of these two data sets leads to the conclusion that both a coordinated inflammatory immune response comprising type 1 CD4^+^ and CD8^+^ T cells, as well as CD14^+^ myeloid cells are potentially required for optimal immune control and clearance of vHSIL. Previously, it has been demonstrated that the rejection of human tissues (tumors, allografts, autoimmune flares) is associated with an almost identical modular signature and defined as the immunological constant of rejection (ICR).^18^, ^19^ In the context of our observations that complete rejections of vHSIL are associated with the presence of vast numbers of type 1 T cells and CD14^+^CD68^−^CD163^−^ myeloid cells, it is of interest to know that the ICR is a process that begins with the induction of a primary inflammatory process leading to the chronic infiltration of monocytes and other cells related to innate immune mechanisms but lack effector function. The presence of pro‐inflammatory signals such as IFNγ, which is likely to come from the Tbet^+^ T cells in vHSIL, induce the activation of monocytes, which in turn, may start to produce pro‐inflammatory cytokines such as IL‐12, IL‐15 and IL‐18, important cytokines to drive and sustain the type 1 immune response required for rejection.^18^, ^19^ Thus, it seems that both imiquimod and therapeutic vaccination will lead to complete rejections when the process of the ICR is already ongoing.

Following this line of reasoning, it also implies that none of the two immunotherapeutic approaches is able to breakdown the barrier existing in cold lesions. We have shown that this is true for therapeutic vaccination.^9^ It is likely that this barrier is induced by the infection with HPV as healthy vulva tissue is strongly infiltrated with immune cells. So what could underlie an impaired primary process of inflammation? We and others have shown that HPV exploits several mechanisms to suppress the primary inflammatory process.^20^, ^21^, ^22^ In addition genomic instability in these lesions^23^, ^24^, ^25^ may turn these lesions cold. In such cases, vHSIL lesions are better treated with cidofovir based on the fact that cidofovir and imiquimod were reported to be effective in two distinct biologically defined vHSIL groups, reflected by their HPV *E2* DNA methylation status.^26^

Our study shows the benefits of using multispectral imaging in order to obtain a profile of the immune microenvironment, which are correlated to the success or failure of an immunotherapeutic treatment. While previous studies^2^, ^7^, ^8^ were unable to find a relation between the numbers of immune infiltrating T cells or myeloid cells and clinical outcome, this was now possible because additional markers allowed for a better definition of the different subpopulations, some of which were correlated with the response to imiquimod or therapeutic vaccination. For future studies, it would be of interest to study the post‐imiquimod immune microenvironment because the imiquimod‐induced changes in the different subsets of immune cells will increase our knowledge on what is exactly required for an effective immune control of HPV‐induced lesions.

## CONFLICT OF INTEREST

The authors declare no potential conflict of interest.

5

## ETHICS STATEMENT

This study was conducted in accordance with the Declaration of Helsinki and approved by the national Central Committee on Research Involving Human Subjects (CCMO, NL21215.000.08) and the local institutional ethical committee (B16.024), which also ruled that no additional informed consent was required according to Dutch law.

## Supporting information


**Table S1** Design seven‐color T‐cell panel and myeloid cell panel, antibodies and detection method per marker
**Table S2.** Subanalyses of the T‐cell panel and myeloid cell panel as used in InForm
**Table S3.** Descriptive statistics of immune cells in vHSIL biopsies, divided based on clinical response, and in healthy vulva
**Figure S1.** Single marker photos of the multiplex, A, T‐cell panel and B, myeloid cell panel in vHSIL
**Figure S2.** InForm training and subanalyses T‐cell panel. The same tissue section is shown in Figure 1A. A, Seven markers signal extraction (Step 1), tissue segmentation (Step 2, epithelium shown in red, stroma shown in green) and cell segmentation (Step 3, cells are marked in light green). B, Division of seven‐color image in four subanalyses for the detection of complex phenotypes: immunofluorescent images (left image), and respective inForm recognition of phenotypes by assigning each phenotype a dot with a specific color (right image). C, The full fluorescent spectrum of the six identified T‐cell phenotypes
**Figure S3**. InForm training and subanalyses myeloid cell panel. The same tissue section is shown in Figure 1B. A, Seven markers signal extraction (Step 1), tissue segmentation (Step 2, epithelium shown in red, stroma shown in green) and cell segmentation (Step 3, cells are marked in light green). B, Division of seven‐color image in three subanalyses for the detection of complex phenotypes: immunofluorescent images (left image), and respective inForm recognition of phenotypes by assigning each phenotype a dot with a specific color (right image). C, The full fluorescent spectrum of the eight identified myeloid cell phenotypes.

## Data Availability

All data generated or analyzed during our study are included in this published article (and its additional files) and are available from the corresponding author on reasonable request.

## References

[1] Hurt CN , Jones S , Madden TA , et al. Recurrence of vulval intraepithelial neoplasia following treatment with cidofovir or imiquimod: results from a multicentre, randomised, phase II trial (RT3VIN). BJOG. 2018;125:1171‐1177. 29336101 10.1111/1471-0528.15124 PMC6055842

[2] van Seters M , van Beurden M , ten Kate FJ , et al. Treatment of vulvar intraepithelial neoplasia with topical imiquimod. N Engl J Med. 2008;358:1465‐1473. 18385498 10.1056/NEJMoa072685

[3] van Poelgeest MI , Welters MJ , Vermeij R , et al. Vaccination against Oncoproteins of HPV16 for noninvasive vulvar/vaginal lesions: lesion clearance is related to the strength of the T‐cell response. Clin Cancer Res. 2016;22:2342‐2350. 26813357 10.1158/1078-0432.CCR-15-2594

[4] Stanley MA . Imiquimod and the imidazoquinolones: mechanism of action and therapeutic potential. Clin Exp Dermatol. 2002;27:571‐577. 12464152 10.1046/j.1365-2230.2002.01151.x

[5] Schon MP , Schon M . Imiquimod: mode of action. Br J Dermatol. 2007;157(Suppl 2):8‐13. 18067624 10.1111/j.1365-2133.2007.08265.x

[6] Schon M , Bong AB , Drewniok C , et al. Tumor‐selective induction of apoptosis and the small‐molecule immune response modifier imiquimod. J Natl Cancer Inst. 2003;95:1138‐1149. 12902443 10.1093/jnci/djg016

[7] Daayana S , Elkord E , Winters U , et al. Phase II trial of imiquimod and HPV therapeutic vaccination in patients with vulval intraepithelial neoplasia. Br J Cancer. 2010;102:1129‐1136. 20234368 10.1038/sj.bjc.6605611 PMC2853099

[8] Winters U , Daayana S , Lear JT , et al. Clinical and immunologic results of a phase II trial of sequential imiquimod and photodynamic therapy for vulval intraepithelial neoplasia. Clin Cancer Res. 2008;14:5292‐5299. 18698049 10.1158/1078-0432.CCR-07-4760

[9] Abdulrahman Z , de Miranda N , van Esch EMG , et al. Pre‐existing inflammatory immune microenvironment predicts the clinical response of vulvar high‐grade squamous intraepithelial lesions to therapeutic HPV16 vaccination. J Immunother Cancer. 2020;8:e000563. 32169871 10.1136/jitc-2020-000563 PMC7069269

[10] Ijsselsteijn ME , Brouwer TP , Abdulrahman Z , et al. Cancer immunophenotyping by seven‐colour multispectral imaging without tyramide signal amplification. J Pathol Clin Res. 2019;5:3‐11. 30191683 10.1002/cjp2.113 PMC6317065

[11] Santegoets S , de Groot AF , Dijkgraaf EM , et al. The blood mMDSC to DC ratio is a sensitive and easy to assess independent predictive factor for epithelial ovarian cancer survival. Onco Targets Ther. 2018;7:e1465166. 10.1080/2162402X.2018.1465166 PMC6136880 30221063

[12] Fridman WH , Zitvogel L , Sautes‐Fridman C , Kroemer G . The immune contexture in cancer prognosis and treatment. Nat Rev Clin Oncol. 2017;14:717‐734. 28741618 10.1038/nrclinonc.2017.101

[13] Gibson SJ , Imbertson LM , Wagner TL , et al. Cellular requirements for cytokine production in response to the immunomodulators imiquimod and S‐27609. J Interferon Cytokine Res. 1995;15:537‐545. 7553223 10.1089/jir.1995.15.537

[14] van Poelgeest MI , van Seters M , van Beurden M , et al. Detection of human papillomavirus (HPV) 16‐specific CD4+ T‐cell immunity in patients with persistent HPV16‐induced vulvar intraepithelial neoplasia in relation to clinical impact of imiquimod treatment. Clin Cancer Res. 2005;11:5273‐5280. 16033846 10.1158/1078-0432.CCR-05-0616

[15] Welters MJ , Kenter GG , de Vos van Steenwijk PJ , et al. Success or failure of vaccination for HPV16‐positive vulvar lesions correlates with kinetics and phenotype of induced T‐cell responses. Proc Natl Acad Sci USA. 2010;107:11895‐11899. 20547850 10.1073/pnas.1006500107 PMC2900675

[16] Welters MJ , van der Sluis TC , van Meir H , et al. Vaccination during myeloid cell depletion by cancer chemotherapy fosters robust T cell responses. Sci Transl Med. 2016;8:334ra52. 10.1126/scitranslmed.aad8307 27075626

[17] Melief CJM , Welters MJ , Vergote I , et al. Strong vaccine responses during chemotherapy are associated with prolonged cancer survival. Sci Transl Med. 2020;12:eaaz8235. 32188726 10.1126/scitranslmed.aaz8235

[18] Wang E , Worschech A , Marincola FM . The immunologic constant of rejection. Trends Immunol. 2008;29:256‐262. 18457994 10.1016/j.it.2008.03.002

[19] Wang E , Monaco A , Monsurró V , et al. Antitumor vaccines, immunotherapy and the immunological constant of rejection. IDrugs. 2009;12:297. 19431094 PMC3410731

[20] Karim R , Tummers B , Meyers C , et al. Human papillomavirus (HPV) upregulates the cellular deubiquitinase UCHL1 to suppress the keratinocyte's innate immune response. PLoS Pathog. 2013;9:e1003384. 23717208 10.1371/journal.ppat.1003384 PMC3662672

[21] Tummers B , Goedemans R , Pelascini LP , et al. The interferon‐related developmental regulator 1 is used by human papillomavirus to suppress NFkappaB activation. Nat Commun. 2015;6:6537. 26055519 10.1038/ncomms7537 PMC4382698

[22] Ma W , Melief CJ , van der Burg SH . Control of immune escaped human papilloma virus is regained after therapeutic vaccination. Curr Opin Virol. 2017;23:16‐22. 28282583 10.1016/j.coviro.2017.02.005

[23] Aulmann S , Schleibaum J , Penzel R , Schirmacher P , Gebauer G , Sinn HP . Gains of chromosome region 3q26 in intraepithelial neoplasia and invasive squamous cell carcinoma of the vulva are frequent and independent of HPV status. J Clin Pathol. 2008;61:1034‐1037. 18559375 10.1136/jcp.2008.056275

[24] Bryndorf T , Kirchhoff M , Larsen J , et al. The most common chromosome aberration detected by high‐resolution comparative genomic hybridization in vulvar intraepithelial neoplasia is not seen in vulvar squamous cell carcinoma. Cytogenet Genome Res. 2004;106:43‐48. 15218240 10.1159/000078559

[25] Rosenthal AN , Ryan A , Hopster D , Surentheran T , Jacobs IJ . High frequency of loss of heterozygosity in vulval intraepithelial neoplasia (VIN) is associated with invasive vulval squamous cell carcinoma (VSCC). Int J Cancer. 2001;94:896‐900. 11745496 10.1002/ijc.1549

[26] Jones SEF , Hibbitts S , Hurt CN , et al. Human papillomavirus DNA methylation predicts response to treatment using Cidofovir and Imiquimod in Vulval intraepithelial Neoplasia 3. Clin Cancer Res. 2017;23:5460‐5468. 28600473 10.1158/1078-0432.CCR-17-0040

