# Supplementary material for: A pre‐existing coordinated inflammatory microenvironment is associated with complete response of vulvar high‐grade squamous intraepithelial lesions to different forms of immunotherapy
Source: Int J Cancer. 2020 Jul 3;147(10):2914–23. doi: 10.1002/ijc.33168 (PMC7540004; doi:10.1002/ijc.33168)
Supplement: Supplementary file 1 — Table S1 Design seven‐color T‐cell panel and myeloid cell panel, antibodies and detection method per marker Table S2. Subanalyses of the T‐cell panel and myeloid cell panel as used in InForm Table S3. Descriptive statistics of immune cells in vHSIL biopsies, divided based on clinical response, and in healthy vulva Figure S1. Single marker photos of the multiplex, A, T‐cell panel and B, myeloid cell panel in vHSIL Figure S2. InForm training and subanalyses T‐cell panel. The same tissue section is shown in Figure 1A. A, Seven markers signal extraction (Step 1), tissue segmentation (Step 2, epithelium shown in red, stroma shown in green) and cell segmentation (Step 3, cells are marked in light green). B, Division of seven‐color image in four subanalyses for the detection of complex phenotypes: immunofluorescent images (left image), and respective inForm recognition of phenotypes by assigning each phenotype a dot with a specific color (right image). C, The full fluorescent spectrum of the six identified T‐cell phenotypes Figure S3. InForm training and subanalyses myeloid cell panel. The same tissue section is shown in Figure 1B. A, Seven markers signal extraction (Step 1), tissue segmentation (Step 2, epithelium shown in red, stroma shown in green) and cell segmentation (Step 3, cells are marked in light green). B, Division of seven‐color image in three subanalyses for the detection of complex phenotypes: immunofluorescent images (left image), and respective inForm recognition of phenotypes by assigning each phenotype a dot with a specific color (right image). C, The full fluorescent spectrum of the eight identified myeloid cell phenotypes. [file IJC-147-2914-s001.pdf]

A pre-existing coordinated inflammatory microenvironment is associated with complete response of vulvar high-grade squamous intraepithelial lesions to different forms of immunotherapy

Ziena Abdulrahman, Noel F.C.C. de Miranda, Bart W.J. Hellebrekers, Peggy J. de Vos van Steenwijk, Edith M.G. van Esch, Sjoerd H. van der Burg, Mariette I.E. van Poelgeest

Supplementary files table of contents:

Supplemental Table 1. Design seven-color T cell panel and myeloid cell panel, antibodies and detection method per marker.

Supplemental Table 2. Sub-analyses of the T cell panel and myeloid cell panel as used in InForm.

Supplemental Table 3. Descriptive statistics of immune cells in vHSIL biopsies, divided based on clinical response, and in healthy vulva.

Supplemental Figure 1. Single marker photos of the multiplex **A)** T cell panel, and **B)** myeloid cell panel in vHSIL.

Supplemental Figure 2. InForm training and sub-analyses T cell panel.

Supplemental Figure 3. InForm training and sub-analyses myeloid cell panel.

Supplemental Table 1. Design seven-color T cell panel and myeloid cell panel, antibodies and detection method per marker.

T cell panel				
**Marker**	**Primary antibody clone and company**	**Primary antibody isotype**	**Secondary antibody**	**Detection method**
**PD-1**	D4W2J Cell Signaling Technology	Rabbit IgG	Poly-HRP and Opal 570	Opal tyramide signal amplification
**Tbet**	D6N8B XP Cell Signaling Technology	Rabbit IgG	Poly-HRP and Opal 520	Opal tyramide signal amplification
**FoxP3**	236A/E7 Invitrogen ThermoFisher	Mouse IgG1	CF goat anti mouse IgG1 633	Secondary antibody fluorochrome labelled
**CD8**	4B11 Novocastra	Mouse IgG2b	Alexa goat anti mouse IgG2b 647	Secondary antibody fluorochrome labelled
**Tim3**	D5D5R XP Cell Signaling Technology	Rabbit IgG	Alexa goat anti rabbit IgG 680	Secondary antibody fluorochrome labelled
**CD3**	D7A6E Cell Signaling Technology, directly labelled with Alexa 594	Rabbit IgG	-	Directly fluorochrome labelled primary antibody
**DAPI**	ThermoFisher	-	-	Fluorescent probe

Myeloid cell panel				
**Marker**	**Primary antibody clone and company**	**Primary antibody isotype**	**Secondary antibody**	**Detection method**
**PD-L1**	SP142 Spring Bioscience	Rabbit IgG	Poly-HRP and Opal 520	Opal tyramide signal amplification
**CD14**	D7A2T Cell Signaling Technology	Rabbit IgG	CF donkey anti rabbit IgG 633	Secondary antibody fluorochrome labelled
**CD33**	PWS44 Novocastra	Mouse IgG2b	Alexa goat anti mouse IgG2b 647	Secondary antibody fluorochrome labelled
**CD163**	10D6 Invitrogen ThermoFisher	Mouse IgG1	CF goat anti mouse IgG1 680	Secondary antibody fluorochrome labelled
**CD11c**	EP1347Y Abcam, directly labelled with Alexa 546	Rabbit IgG	-	Directly fluorochrome labelled primary antibody
**CD68**	D4B9C XP Cell Signaling Technology, directly labelled with Alexa 594	Rabbit IgG	-	Directly fluorochrome labelled primary antibody
**DAPI**	ThermoFisher	-	-	Fluorescent probe

Supplemental Table 2. Sub-analyses of the T cell panel and myeloid cell panel as used in InForm.

	**T cell panel**		**Myeloid cell panel**	
**Sub-analysis 1**	CD3$^+$CD8$^-$ FoxP3$^-$	(T helper cell)	CD68$^+$CD163$^-$	(M1 macrophage)
	CD3$^+$CD8$^+$FoxP3$^-$	(CD8$^+$ T cell)	CD68$^+$CD163$^+$	(M2 macrophage)
	CD3$^+$CD8$^-$ FoxP3$^+$	(regulatory T cell)	CD68$^-$CD163$^+$	(CD163$^+$ non-macrophage)
	CD3$^-$CD8$^-$FoxP3$^-$	(no T cell)	CD68$^-$CD163$^-$	(non-macrophage)
**Sub-analysis 2**	Tbet$^+$	(IFNγ producing T cell)	CD11c$^+$CD14$^-$	(DC)
	Tbet$^-$	(Tbet$^-$ cell)	CD11c$^+$CD14$^+$	(inflammatory DC)
			CD11c$^-$CD14$^+$	(inflammatory myeloid cell)
			CD11c$^-$CD14$^-$	(CD11c$^-$ and CD14$^-$ cell)
**Sub-analysis 3**	PD1$^+$	(activated PD1$^+$ T cell)	CD33$^+$PDL1$^-$	(immature myeloid cell)
	PD1$^-$	(PD1$^-$ cell)	CD33$^+$PDL1$^+$	(immature PDL1$^+$ myeloid cell)
			CD33$^-$PDL1$^+$	(mature PDL1$^+$ myeloid cell)
			CD33$^-$PDL1$^-$	(CD33$^-$ and PDL1$^-$ cell)
**Sub-analysis 4**	Tim3$^+$	(activated Tim3$^+$ T cell)		
	Tim3$^-$	(Tim3$^-$ cell)		

Supplemental Table 3. Descriptive statistics of immune cells in vHSIL biopsies, divided based on clinical response, and in healthy vulva.

Immune cell phenotype	Number of cells/mm² epithelium (median with 95% CI)					Number of cells/mm² stroma (median with 95% CI)				
	NR	PR	CR	Healthy	Statistical significant difference	NR	PR	CR	Healthy	Statistical significant difference
$CD3^+CD8^-$	4.97 (0-12.07)	6.56 (0-25.21)	3.62 (1.55-14.45)	9.53 (3.08-26.57)	-	6.86 (2.49-24.02)	20.46 (1.68-38.59)	21.47 (3.58-159.30)	22.92 (9.76-59.35)	-
$CD3^+CD8^-TBET^+$	11.47 (0.76-18.29)	11.57 (2.09-24.42)	11.24 (2.06-44.51)	28.74 (8.04-52.88)	-	45.74 (8.71-166.9)	41.57 (12.47-91.48)	92.05 (22.47-177.6)	40.39 (20.09-76.78)	-
$CD3^+CD8^-PD1^+TBET^+$	1.64 (0-2.93)	0.61 (0-10.31)	0.98 (0-10.39)	8.09 (2.02-15.73)	PR*H, CR*H	7.27 (0.41-11.43)	8.43 (0-30.35)	15.70 (0.43-58.20)	10.66 (3.28-20.19)	-
$CD3^+CD8^-FOXP3^+$	0.73 (0-0.76)	3.68 (0.91-5.17)	4.69 (0.20-8.26)	0.43 (0-2.86)	PR*H, CR*H	18.29 (1.45-83.14)	35.18 (7.12-61.34)	59.73 (3.90-211.90)	2.91 (0.98-6.73)	PR****H, CR***H
$CD3^+CD8^+$	-	-	-	-	-	3.43 (1.45-106.2)	35.90 (28.49-110.8)	43.32 (9.42-105.4)	10.29 (5.27-14.30)	PR**H, CR**H
$CD3^+CD8^+TBET^+$	-	-	-	-	-	13.72 (0.62-172.3)	16.89 (6.15-50.44)	22.18 (3.77-99.17)	10.21 (3.66-26.75)	-
$CD14^+CD68^-CD163^-$	22.21 (0.18-82.93)	3.87 (0-63.56)	11.77 (0-41.62)	36.20 (14.35-57.23)	-	16.71 (16.68-360.3)	62.41 (15.80-183.90)	84.25 (50.19-386.80)	138.10 (88.72-239.10)	PR*H
$CD14^+CD68^+CD163^-$	-	-	-	-	-	37.88 (0-70.83)	8.88 (0.37-37.66)	9.04 (1.21-80.05)	24.85 (11.03-49.57)	PR*H
$CD14^+CD68^+CD163^+$	-	-	-	-	-	29.60 (1.85-212.5)	17.08 (4.28-32.44)	42.38 (16.96-59.32)	62.99 (25.63-89.09)	PR*CR, PR**H
$CD14^-CD68^+CD163^-$	4.15 (0-22.21)	0.82 (0-12.21)	10.62 (1.33-32.04)	1.26 (0-3.24)	CR**H	55.70 (0-108.2)	6.73 (1.10-55.87)	28.09 (1.21-145.6)	11.42 (4.98-18.77)	-
$CD14^-CD68^-CD163^+$	-	-	-	-	-	5.56 (0-7.73)	0.72 (0-138.00)	6.07 (0.27-19.45)	2.39 (0.36-5.86)	-
$CD14^-CD68^+CD163^+$	-	-	-	-	-	9.87 (7.41-37.67)	11.49 (0-18.02)	9.95 (4.57-19.99)	9.07 (2.32-18.42)	-
$CD14^-CD68^-CD163^-CD33^+$	33.14 (15.16-73.72)	5.81 (2.24-22.54)	9.93 (7.15-19.56)	8.08 (2.97-16.08)	-	74.12 (16.55-177.70)	34.48 (16.92-88.76)	36.74 (9.74-72.39)	23.90 (8.84-41.58)	-
$CD14^-CD68^-CD163^-CD11c^+$	-	-	-	-	-	2.55 (0-6.12)	6.63 (1.44-11.53)	5.26 (0.99-12.07)	0.95 (0.27-4.71)	PR*H, CR*H

Non-responders (NR, n=3), partial responders (PR, n=12), complete responders (CR, n=14) and healthy vulva (H, n=27). A threshold of a median cell count of ≥10 cells/mm² per tissue compartment was applied, to only select relevant phenotypes for analyses. NRs have not been included in the statistical analyses, due to their small sample size. Differences between two groups were calculated with a Mann-Whitney test, statistical significance is indicated with asterisks: *p<0.05, **p<0.01, ***p<0.001 and ****p<0.0001.

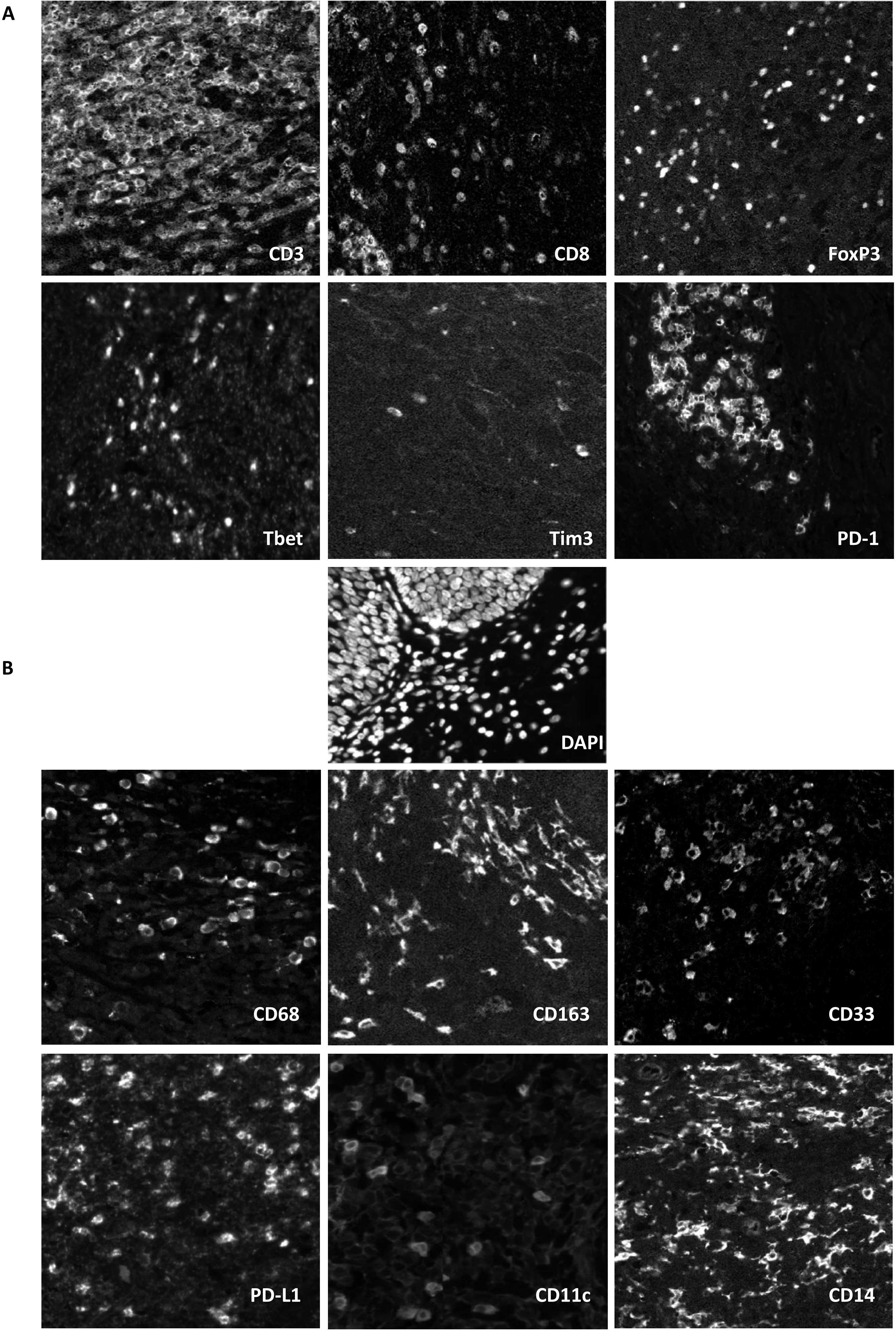

Supplemental Figure 1, Abdulrahman et al.

Single marker photos of the multiplex **A)** T cell panel and **B)** myeloid cell panel in vHSIL.

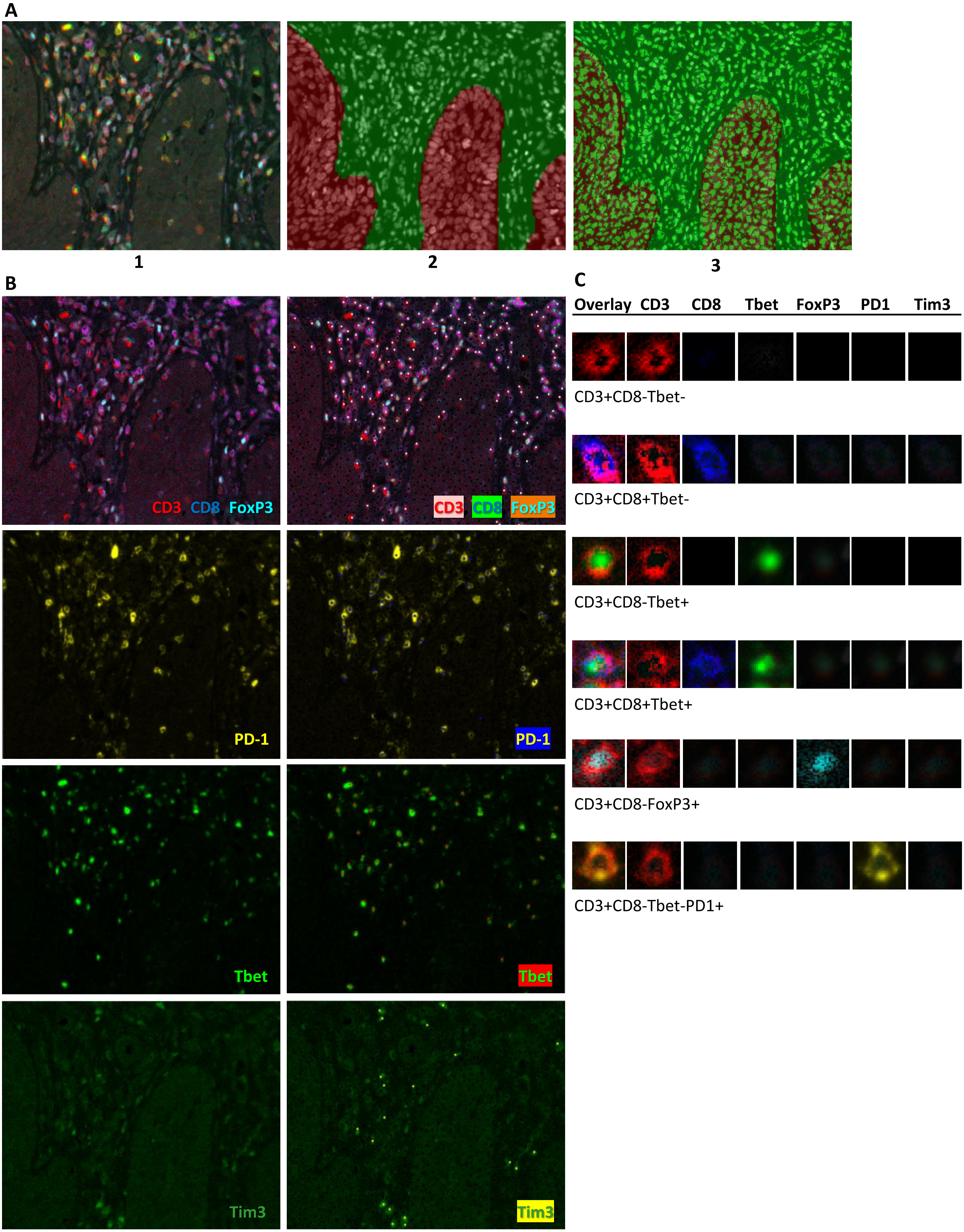

Supplemental Figure 2, Abdulrahman et al. InForm training and sub-analyses T cell panel. The same tissue section is shown in Figure 1A. **A)** Seven markers signal extraction (step 1), tissue segmentation (step 2) and cell segmentation (step 3). **B)** Division of seven-color image in four sub-analyses for the detection of complex phenotypes: immunofluorescent images (left image), and respective inForm recognition of phenotypes by assigning each phenotype a dot with a specific color (right image). **C)** The full fluorescent spectrum of the six identified T cell phenotypes.

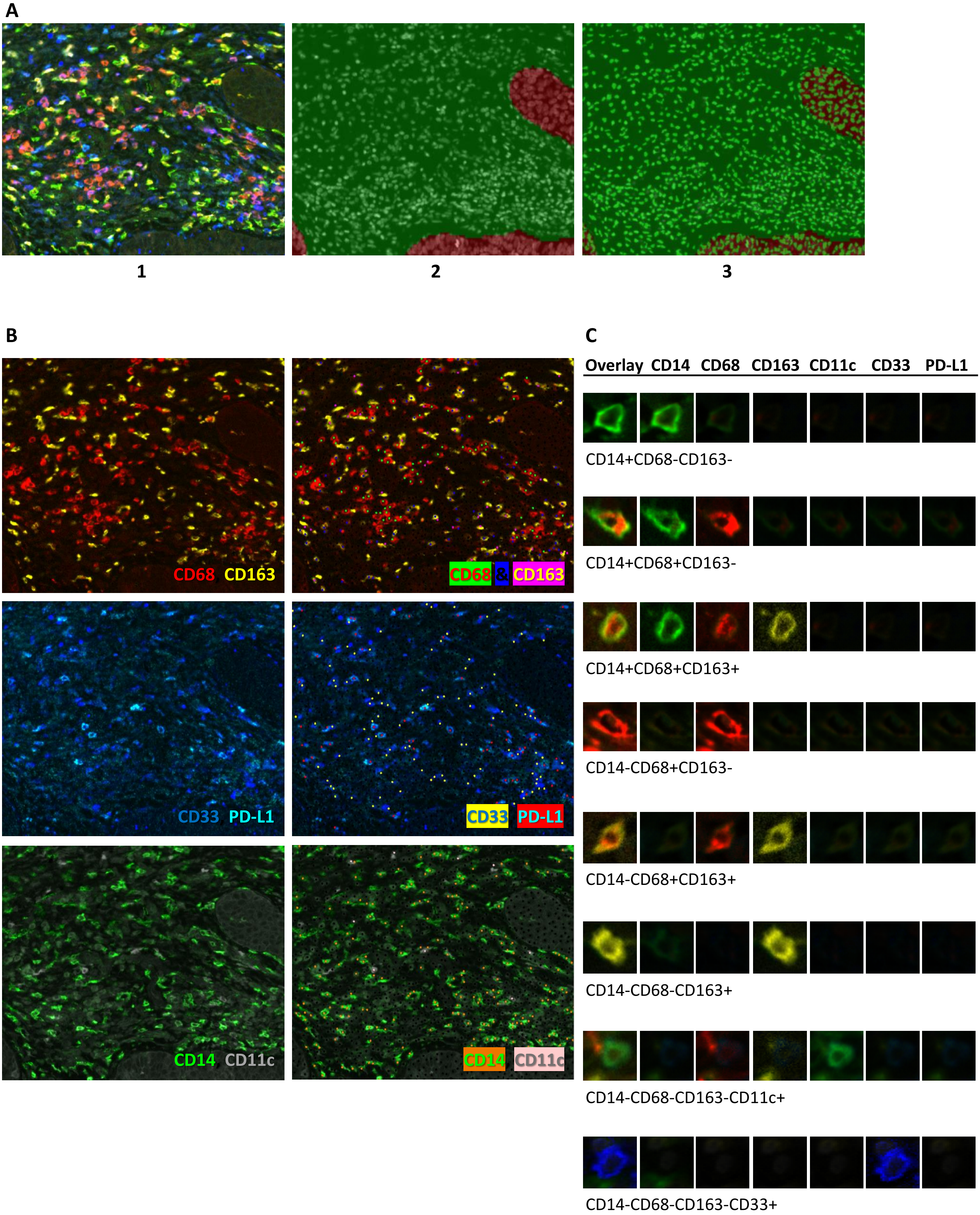

Supplemental Figure 3, Abdulrahman et al. InForm training and sub-analyses myeloid cell panel. The same tissue section is shown in Figure 1B. **A)** Seven markers signal extraction (step 1), tissue segmentation (step 2) and cell segmentation (step 3). **B)** Division of seven-color image in three sub-analyses for the detection of complex phenotypes: immunofluorescent images (left image), and respective inForm recognition of phenotypes by assigning each phenotype a dot with a specific color (right image). **C)** The full fluorescent spectrum of the eight identified myeloid cell phenotypes.
